# Advances in Research on *Epichloë* endophytes in Chinese Native Grasses

**DOI:** 10.3389/fmicb.2016.01399

**Published:** 2016-09-07

**Authors:** Hui Song, Zhibiao Nan, Qiuyan Song, Chao Xia, Xiuzhang Li, Xiang Yao, Wenbo Xu, Yu Kuang, Pei Tian, Qingping Zhang

**Affiliations:** State Key Laboratory of Grassland Agro-Ecosystems, College of Pastoral Agriculture Science and Technology, Lanzhou UniversityLanzhou, China

**Keywords:** alkaloid, Chinese native grass, *Epichloë* endophyte, grass-*Epichloë* symbiosis, grass stress response, *Epichloë* phylogeny

## Abstract

*Epichloë* fungal endophytes are broadly found in cool-season grasses. The symbiosis between these grasses and *Epichloë* may improve the abiotic and biotic resistance of the grass plant, but some *Epichloë* species produce alkaloids that are toxic for livestock. Therefore, it is important to understand the characteristics of the grass-*Epichloë* s symbiosis so that the beneficial aspects can be preserved and the toxic effects to livestock can be avoided. Since the 1990s, Chinese researchers have conducted a series of studies on grass-*Epichloë* symbiosis. In this review, we describe the current state of *Epichloë* endophyte research in Chinese native grasses. We found that more than 77 species of native grasses in China are associated with *Epichloë* endophytes. In addition, we review the effects of various *Epichloë* species on native grass responses to abiotic and biotic stress, phylogeny, and alkaloid production. We provide an overview of the study of *Epichloë* species on native grasses in China and directions for future research.

## Introduction

Fungi of the genus *Epichloë* (Clavicipitaceae, Ascomycota) and their asexual state (*Neotyphodium*) are common endophytes of cool-season grasses in the subfamily Pooideae (Leuchtmann et al., 2014). Most previous research has indicated that asexual *Epichloë* species (29 species) are efficiently transmitted through host seeds (vertical transmission) (White et al., 1993; Leuchtmann et al., 2014). However, some recent studies have suggested that strictly asexual *Epichloë* endophytes are occasionally transmitted horizontally among plants in close proximity via frequent mowing, trampling, and grazing (Cheplick and Faeth, 2009; Iannone et al., 2009; Wiewióra et al., 2015; Saikkonen et al., 2016), and by conidia from epiphytic mycelia (Tadych et al., 2007, 2012; Oberhofer and Leuchtmann, 2014) via splashing water and possibly wind. Sexual *Epichloë* species (12 species) are transmitted to new hosts with filamentuos ascospores in addition to condia (horizontal transmission) (Leuchtmann et al., 2014; Saikkonen et al., 2016). Leuchtmann et al. (2014) renamed the anamorphs of *Neotyphodium* as the asexual endophyte genus *Epichloë* and examined the classification of sexual and asexual *Epichloë* species and varieties based on β-tubulin (*tubB*) sequences.

*Epichloë* species often provide numerous benefits to their hosts, such as increased tolerance to drought (Malinowski and Belesky, 2000; Kannadan and Rudgers, 2008; Gundel et al., 2013b), disease resistance (Vignale et al., 2013), resistance to herbivory and parasitism (Bush et al., 1997; Schardl et al., 2007; Gundel et al., 2013a), and enhanced aboveground and belowground vegetative and reproductive growth (Marks et al., 1991; Clay and Holah, 1999; Yue et al., 2000; Gundel et al., 2013b; Tadych et al., 2014). Previous studies have confirmed that certain alkaloids play a crucial role in a plant's pasture persistence. For example, lolines and peramine may confer significant toxicity against insect pests (Johnson et al., 1985, 2013; Schardl et al., 2013; Philippe, 2016). However, conflicting results have been reported. When *Lolium perenne* was grown under conditions of extremely poor nutrient availability, *Epichloë festucae* var. *lolii* infection led to a reduced root: shoot ratio and reduced photosynthetic shoot fraction (Cheplick, 2007). Some symbiont combinations, such as *Schedonorus arundinaceus* with *Epichloë coenophiala* and *Lolium perenne* with *E. festucae* var. *lolii*, accumulate alkaloids that are toxic to grazing animals (Di Menna et al., 2012; Schardl et al., 2013; Philippe, 2016). On the other hand, sexual *Epichloë* species could result in “choke disease” in host plants, in which sexual *Epichloë* species produce stromata that envelop the inflorescences and upper leaf sheaths of flowering culms; this leads to a reduced number of offspring (Lembicz et al., 2010).

Various *Epichloë* species have been discovered in China, but have not been formally taxonomically described. There are two reasons for this lack of taxonomic data: (i) the limited number of researchers in this field and (ii) insufficient knowledge on the identification and classification of *Epichloë* species. To address the latter issue, Chinese researchers are establishing collaborations with international institutes. The topic of hybrid occurrence in Chinese *Epichloë* species is not discussed in-depth in this manuscript because few *Epichloë* species are confirmed to be of hybrid origin. However, known hybrid species from native grasses appear to have the same two ancestors, for two main reasons. First, researchers have only confirmed some *Epichloë* species crosses for the *Epichloë bromicola* × *Epichloë typhina* complex. Second, hybrid species are distributed in the same and similar natural and geographic environments. Accordingly, these hybrid species underwent the same hybridization process, but are hosted by different grasses. This topic will be discussed in future reviews when more data are available on hybrid endophytes.

We have built a long-term collaboration with Prof. Christopher L. Schardl from the University of Kentucky and Prof. German Spangenberg from the Australian Academy of Technological Sciences and Engineering. With their help, two kinds of *Epichloë* endophytes in drunken horse grass were confirmed. The whole genome sequencing of an *Epichloë* endophyte in *Festuca sinensis* is near completion. These studies will push *Epichloë* research to a new level in China. We firmly believe that the research prospects with respect to *Epichloë* species are bright in our country.

## The distribution and diversity of grass-*Epichloë* symbiosis

**Figure 1:**
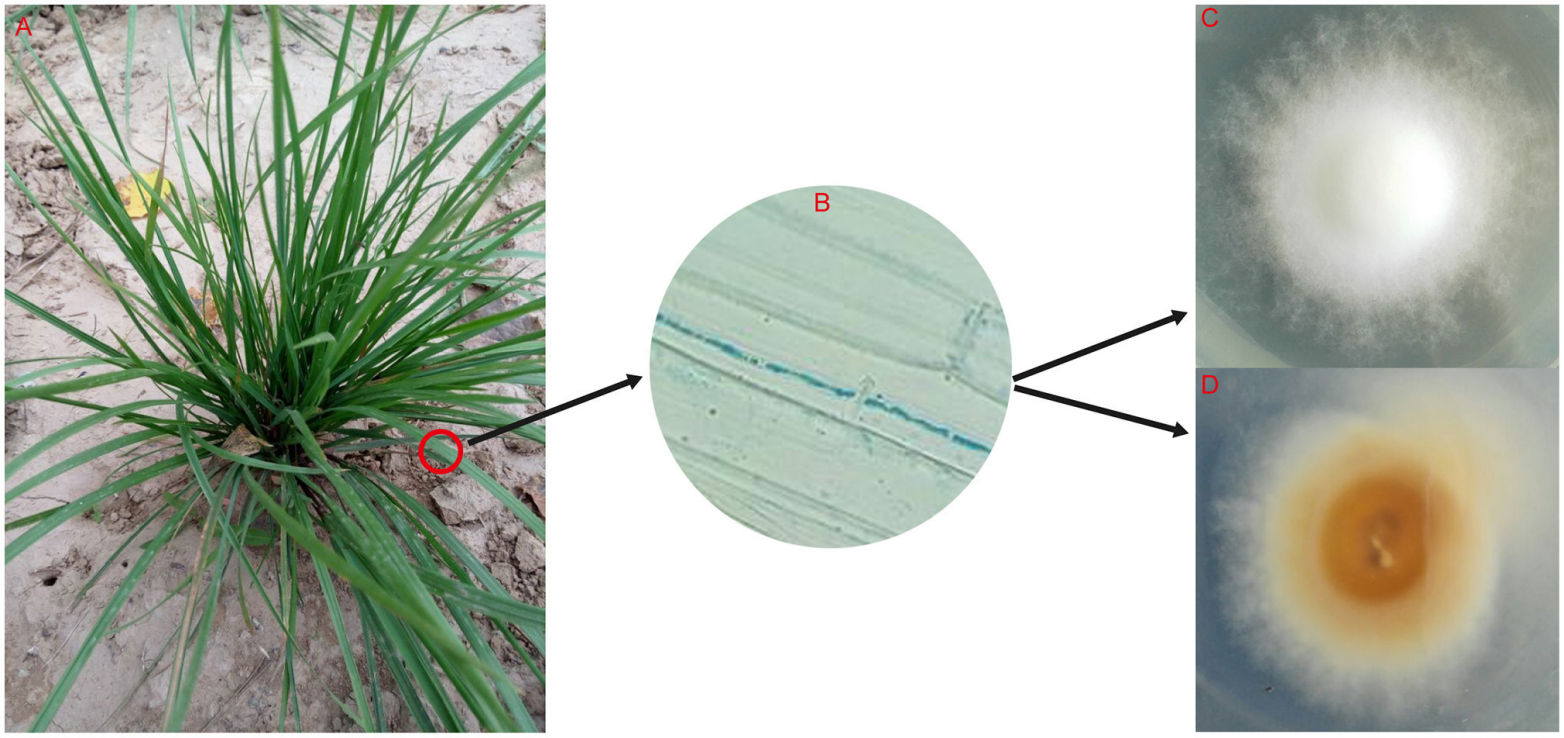
**Identification and isolation of ***Epichloë*** endophytes in ***Festuca sinensis***. (A)** *Festuca sinensis*. **(B)** Microscope image of hyphae. Blue line indicates hyphae of *Epichloë* endophyte detected using the aniline coloring (0.1% aqueous) method, isolated on potato dextrose agar, and incubated in the dark at 25°C for 4 weeks. **(C)** Obverse of colony. **(D)** Reverse of colony.

More than 77 species of native grasses in China have been documented as infected with *Epichloë* species (Nan and Li, 2000; Li et al., 2004, 2006b, 2009, 2012b; Wang et al., 2005; Wei et al., 2006; Moon et al., 2007; Chen et al., 2009; Ji et al., 2009, 2011, 2012; Kang et al., 2009, 2011a; Zhan et al., 2009; Zhang et al., 2009, 2011a, 2013; Han et al., 2012; Zhu et al., 2013; Card et al., 2014; Leuchtmann et al., 2014). The endophytes have been found in the following grass genera: *Achnatherum, Agropyron, Agrostis, Brachypodium, Bromus, Calamagrostis, Cleistogenes, Dactylis, Deschampsia, Elymus, Elytrigia, Eragrostis, Festuca, Hordeum, Koeleria, Leymus, Melica, Poa, Polypopon, Roegneria*, and *Stipa* (Table 1). Among these, many species of Triticeae, Stipeae, and Poeae have been reported as infected and some new *Epichloë* species have been described from these tribes (Li et al., 2004, 2006b; Wei et al., 2006; Chen et al., 2009; Kang et al., 2009, 2011a; Zhu et al., 2013). To date, nine *Epichloë* species have been identified from Chinese native grasses (Li et al., 2009; Leuchtmann et al., 2014). Unfortunately, many isolates from Chinese native grasses have not been identified to the species level based on morphology and DNA data (Table 1). For example, an *Epichloë* endophyte was isolated from *Festuca sinensis* (Figure 1). We found that this *Epichloë* endophyte is likely a new species, based on phylogenetic trees constructed using many markers. However, this research is still in progress. We posit that many *Epichloë* species new to science could be infecting Chinese native grasses.

**Table 1:** **Summary of ***Epichloë*** endophytes in Chinese native grasses**.

**Endophyte**	**Isolate**	**Host species**	**Host tribe**	**Hybrid status**	**Sexual reproduction**	**References**
*Epichloë bromicola*	Yes	*Brachypodium sylvaticum*	Brachypodieae	Not observed	Observed	Ji et al., 2011, 2012
*Epichloë bromicola*	Yes	*Bromus magnus*	Bromeae	Non-hybrid	Not observed	Zhang et al., 2013
*Epichloë bromicola*	Yes	*Elymus dahuricus*	Triticeae	Non-hybrid	Not observed	Song and Nan, 2015
*Epichloë bromicola*	Yes	*Elymus dahuricus* var. *cylindricus*	Triticeae	Non-hybrid	Not observed	Song and Nan, 2015
*Epichloë bromicola*	Yes	*Elymus excelsus*	Triticeae	Non-hybrid	Not observed	Song and Nan, 2015
*Epichloë bromicola*	Yes	*Elymus nutans*	Triticeae	Non-hybrid	Not observed	Song and Nan, 2015
*Epichloë bromicola*	Yes	*Elymus tangutorum*	Triticeae	Non-hybrid	Not observed	Song and Nan, 2015
*Epichloë bromicola*	Yes	*Elymus tibeticus*	Triticeae	Non-hybrid	Not observed	Song and Nan, 2015
*Epichloë bromicola*	Yes	*Leymus chinensis*	Triticeae	Non-hybrid	Not observed	Wei et al., 2006; Zhu et al., 2013
*Epichloë bromicola*	Yes	*Roegneria kamoji*	Triticeae	Non-hybrid	Observed	Li et al., 2006b
*Epichloë gansuensis*	Yes	*Achnatherum inebrians*	Stipeae	Non-hybrid	Not observed	Nan and Li, 2000; Li et al., 2004
*Epichloë gansuensis*	Yes	*Achnatherum pekinense*	Stipeae	Non-hybrid	Not observed	Leuchtmann et al., 2014
*Epichloë gansuensis* var. *inebrians*	Yes	*Achnatherum inebrians*	Stipeae	Non-hybrid	Not observed	Moon et al., 2007
*Epichloë liyangensis*	Yes	*Poa pratensis* ssp. *pratensis*	Poeae	Hybrid–*Epichloë bromicola* x *Epichloë typhina* complex	Observed	Kang et al., 2011a
*Epichloë sibirica*	Yes	*Achnatherum sibiricum*	Stipeae	Non-hybrid	Not observed	Zhang et al., 2009
*Epichloë sinica*	Yes	*Roegneria* spp.	Triticeae	Hybrid–*Epichloë bromicola* x *Epichloë typhina* complex	Not observed	Kang et al., 2009
*Epichloë sinofestucae*	Yes	*Festuca parvigluma*	Poeae	Hybrid–*Epichloë bromicola* x *Epichloë typhina* complex	Not observed	Chen et al., 2009
*Epichloë stromatolonga*	Yes	*Calamagrostis epigeios*	Aveneae	Non-hybrid	Not observed	Ji et al., 2009
*Epichloë typhina*	Yes	*Dactylis glomerata*	Poaeae	Non-hybrid	Observed	Li et al., 2009
*Epichloë* sp.	No	*Achnatherum purpurascens*	Stipeae	Not observed	Not observed	Wei et al., 2006
*Epichloë* sp.	No	*Achnatherum splendens*	Stipeae	Not observed	Not observed	Nan and Li, 2000; Wei et al., 2006
*Epichloë* sp.	No	*Agropyron cirstatum* cvr. *pectiniforme*	Triticeae	Not observed	Not observed	Wei et al., 2006
*Epichloë* sp.	No	*Agropyron cristatum*	Triticeae	Not observed	Not observed	Nan and Li, 2000
*Epichloë* sp.	No	*Agropyron desertorum*	Triticeae	Not observed	Not observed	Wei et al., 2006
*Epichloë* sp.	No	*Agropyron elongata*	Triticeae	Not observed	Not observed	Wei et al., 2006
*Epichloë* sp.	No	*Agropyron mongolicum*	Triticeae	Not observed	Not observed	Wei et al., 2006
*Epichloë* sp.	Yes	*Agrostis* sp	Aveneae	Not observed	Not observed	Ji et al., 2011
*Epichloë* sp.	Yes	*Agrostis* spp	Aveneae	Not observed	Not observed	Wang et al., 2005
*Epichloë* sp.	No	*Bromus inermis*	Bromeae	Not observed	Not observed	Nan and Li, 2000
*Epichloë* sp.	Yes	*Bromus* sp.	Bromeae	Not observed	Not observed	Ji et al., 2011
*Epichloë* sp.	Yes	*Bromus* spp.	Bromeae	Not observed	Not observed	Wang et al., 2005
*Epichloë* sp.	Yes	*Calamagrostis* sp.	Aveneae	Not observed	Not observed	Zhan et al., 2009
*Epichloë* sp.	No	*Cleistogenes squarrosa*	Eragrostideae	Not observed	Not observed	Wei et al., 2006
*Epichloë* sp.	No	*Deschampsia caespitosa*	Aveneae	Not observed	Not observed	Nan and Li, 2000
*Epichloë* sp.	Yes	*Elymus ciliaris*	Triticeae	Not observed	Not observed	Card et al., 2014
*Epichloë* sp.	No	*Elymus cylindricus*	Triticeae	Not observed	Not observed	Nan and Li, 2000
*Epichloë sp*.	Yes	*Elymus dahuricus* var. *excelsus*	Triticeae	Not observed	Not observed	Card et al., 2014
*Epichloë* sp.	Yes	*Elymus gmelinii*	Triticeae	Not observed	Not observed	Card et al., 2014
*Epichloë* sp.	Yes	*Elymus nevskii*	Triticeae	Not observed	Not observed	Card et al., 2014
*Epichloë* sp.	Yes	*Elymus sibiricus*	Triticeae	Not observed	Not observed	Wei et al., 2006; Card et al., 2014
*Epichloë* sp.	Yes	*Elymus* sp.	Triticeae	Not observed	Not observed	Nan and Li, 2000
*Epichloë* sp.	No	*Elytrigia dahuricus*	Triticeae	Not observed	Not observed	Nan and Li, 2000
*Epichloë* sp.	No	*Elytrigia repens*	Triticeae	Not observed	Not observed	Wei et al., 2006
*Epichloë* sp.	No	*Elytrigia smitihii*	Triticeae	Not observed	Not observed	Wei et al., 2006
*Epichloë* sp.	Yes	*Eragrostis pilosa*	Eragrostideae	Not observed	Not observed	Ji et al., 2011
*Epichloë* sp.	No	*Festuca alatavica*	Poeae	Not observed	Not observed	Nan and Li, 2000
*Epichloë* sp.	No	*Festuca modesta*	Poeae	Not observed	Not observed	Nan and Li, 2000
*Epichloë* sp.	Yes	*Festuca myuros*	Poeae	Hybrid–*Epichloëbromicola x Epichloë typhina* complex	Not observed	Han et al., 2012
*Epichloë* sp.	No	*Festuca rubra*	Poeae	Not observed	Not observed	Nan and Li, 2000
*Epichloë* sp.	Yes	*Festuca sinensis*	Poeae	Not observed	Not observed	Nan and Li, 2000
*Epichloë* sp.	Yes	*Festuca* sp.	Poeae	Not observed	Not observed	Ji et al., 2011
*Epichloë* sp.	Yes	*Festuca* spp.	Poeae	Not observed	Not observed	Wang et al., 2005
*Epichloë* sp.	Yes	*Hordeum bogdanii*	Triticeae	Not observed	Not observed	Nan and Li, 2000
*Epichloë* sp.	Yes	*Hordeum brevisubulatum*	Triticeae	Not observed	Not observed	Nan and Li, 2000; Wei et al., 2006
*Epichloë* sp.	Yes	*Hordeum jubatum*	Triticeae	Not observed	Not observed	Unpublished data
*Epichloë* sp.	Yes	*Hordeum roshevitzii*	Triticeae	Not observed	Not observed	Card et al., 2014
*Epichloë* sp.	No	*Hordeum violaceum*	Triticeae	Not observed	Not observed	Nan and Li, 2000
*Epichloë* sp.	No	*Koeleria cristata*	Aveneae	Not observed	Not observed	Wei et al., 2006
*Epichloë* sp.	Yes	*Melica przewalskyi*	Meliceae	Not observed	Not observed	Li et al., 2012b
*Epichloë* sp.	No	*Poa alpina*	Poeae	Not observed	Not observed	Nan and Li, 2000
*Epichloë* sp.	No	*Poa angustifolia*	Poeae	Not observed	Not observed	Wei et al., 2006
*Epichloë* sp.	No	*Poa annua*	Poeae	Not observed	Not observed	Wei et al., 2006
*Epichloë* sp.	No	*Poa palustris*	Poeae	Not observed	Not observed	Wei et al., 2006
*Epichloë* sp.	No	*Poa paucifolia*	Poeae	Not observed	Not observed	Wei et al., 2006
*Epichloë* sp.	No	*Poa pratensis*	Poeae	Not observed	Not observed	Wei et al., 2006
*Epichloë* sp.	Yes	*Poa* spp.	Poeae	Not observed	Not observed	Ji et al., 2011
*Epichloë* sp.	No	*Poa sphondylodes*	Poeae	Not observed	Not observed	Nan and Li, 2000
*Epichloë* sp.	No	*Poa tibetan*	Poeae	Not observed	Not observed	Nan and Li, 2000
*Epichloë* sp.	No	*Polypogon monspeliensis*	Agrostideae	Not observed	Not observed	Nan and Li, 2000
*Epichloë* sp.	Yes	*Roegneria canina*	Triticeae	Not observed	Not observed	Zhang et al., 2011a
*Epichloë* sp.	Yes	*Roegneria ciliaris*	Triticeae	Not observed	Not observed	Wang et al., 2005
*Epichloë* sp.	Yes	*Roegneria hybrida*	Triticeae	Not observed	Not observed	Wang et al., 2005
*Epichloë* sp.	Yes	*Roegneria mayebarana*	Triticeae	Not observed	Not observed	Wang et al., 2005
*Epichloë* sp.	No	*Roegneria stricta*	Triticeae	Not observed	Not observed	Nan and Li, 2000
*Epichloë* sp.	No	*Roegneria turczaninovii*	Triticeae	Not observed	Not observed	Wei et al., 2006
*Epichloë* sp.	No	*Stipa grandis*	Stipeae	Not observed	Not observed	Wei et al., 2006
*Epichloë* sp.	No	*Stipa purpurea*	Stipeae	Not observed	Not observed	Nan and Li, 2000

Most *Epichloë* species are asexual endophytes without external symptoms in their Chinese host grasses (Leuchtmann et al., 2014), such as *E. bromicola, E. gansuensis, E. gansuensis* var. *inebrians, E. sibirica*, and *E. sinica*. However, *Dactylis glomerata* (Li et al., 2009), *Roegneria kamoji* (Li et al., 2006b), and *Poa pratensis* ssp. *pratensis* (Kang et al., 2011a) can also be infected with sexual *Epichloë* species. Although most Chinese *Epichloë* endophytes are not hybrids, *E. sinofestucae* (from *F. parvigluma*) (Chen et al., 2009), *E. sinica* (from *Roegneria* spp.) (Kang et al., 2009), *E. liyangensis* (from *P. pratensis* ssp. *pratensis*) (Kang et al., 2011a), and *E*. sp. (from *F. myuros*) (Han et al., 2012) are hybrids of *E. bromicola* and *E. typhina* (Table 1). *E. bromicola i*s abundant in its host genera *Elymus, Hordeum*, and *Roegneria*, including some of the most widely distributed grass species native to China. The hybrid species *E. liyangensis, E. sinica, E. sinofestucae*, and other *Epichloë* spp. have a common ancestor, e.g., the sexual *E. bromicola* from *R. kamoji* in China.

Interestingly, *Epichloë* endophytes in natural grasses are morphologically diverse, e.g., the species that infect *Achnatherum sibiricum* (Wei et al., 2007) and *Elymus* species (Song et al., 2015b). Ren et al. (2009) isolated 484 *Epichloë* endophytes from seven populations of *A. sibiricum* in Inner Mongolia, China and detected five morphotypes that also exhibited different magnitudes of inhibition of *Rhizoctonia solani, Fusarium oxysporum, Curvularia lunata, Cladosporium cucumerium*, and *Phomopsis vexans*. Researchers have also detected morphological differences along an altitudinal gradient. *Epichloë* isolates from populations of *Elymus* above 3000 m present similar morphological traits, while *Epichloë* populations below 3000 m are morphologically variable (Song et al., 2015b). Asexual *Epichloë* endophytes below 3000 m tend to grow faster on potato dextrose agar than asexual *Epichloë* endophytes above 3000 m (Song et al., 2015b). In addition, a phylogenetic analysis showed that *Epichloë* endophytes above 3000 m form a clade, but isolates from regions below 3000 m belong to several clades (Song et al., 2015b).

## Effects of *Epichloë* species on abiotic and biotic stress in grasses

### Salt stress

Plant cells are harmed by salt stress and do not intake sodium as an essential element for their physiology. Although plants have evolved several strategies to adapt to salt stress (Zhu, 2003; Dinneny, 2015), only a few studies have confirmed that *Epichloë* endophytes can increase salt tolerance in a grass host (Reza Sabzalian and Mirlohi, 2010). When *Hordeum brevisubulatum* was infected with *Epichloë* (EI), the grass exhibited significantly increased N, P, and K^+^ concentrations, which led to an increase in total biomass. The *Epichloë* infection also reduced Na^+^ accumulation in the EI plants compared to *Epichloë*-free plants (EF) (Song et al., 2015d). Based on this work, we inferred that salt tolerance could be further increased in grass-*Epichloë* symbiosis, which potentially provide a valuable resource for improved salt tolerance in crops.

### Drought stress

Compared to salt stress, crop plants are inclined to suffer from drought stress (Boyer, 1982). Studies have confirmed *Epichloë* endophytes play a vital role in increasing drought tolerance in EI grasses (Richardson et al., 1992; Clay and Schardl, 2002; Schardl et al., 2004). A relationship between increased drought tolerance and EI has been well documented in five EI grasses that are native to China. Under drought stress, EI *Leymus chinensis* had significantly more total biomass than EF *L. chinensis*, regardless of fertilizer levels (Ren et al., 2014). Peng et al. (2013) found that seed hydropriming treatment is an effective strategy to improve seed germination and plant growth in EI *F. sinensis*. *Epichloë* infection also increased the germination of *Elymus dahuricus* under different osmotic potential pressures, but germination success was variable among populations (Zhang and Nan, 2010). Several studies have shown that *Epichloë* infection can improve the relative fitness of grasses under drought stress (Faeth, 2002; Faeth et al., 2004; Iannone et al., 2012). Zhang and Nan (2007b) showed that EI *E. dahuricus* produced more biomass, more tillers, and taller plants under low water treatment, but EI had no influence on plant biomass in the high water treatment. However, in a study of EI *A. sibiricum*, the addition of fertilizer resulted in greater plant growth, but this advantage decreased under reduced water and/or nutrient availability (Ren et al., 2011). Moreover, Song et al. (2015e) demonstrated that asexual *Epichloë* endophyte infection can increase resistance to waterlogging stress in *H. brevisubulatum*. The effect of EI on drought tolerance seems to differ among grass species. It remains to be determined whether these effects are caused by the species of infectious *Epichloë*, the grass species, or other factors.

### Other abiotic stress

*Epichloë* endophytes confer stress tolerance to native grasses in China and play a significant role in the survival of some plants in high-stress environments, such as cadmium (Cd)-contaminated soils and nutrient-depleted soils. *Epichloë*-infected *A. inebrians* (Zhang et al., 2010a,b) and *E. dahuricus* (Zhang et al., 2012a) had higher germination rates, more tillers, longer shoots and roots, and more biomass compared to EF plants in high Cd^2+^ concentrations. There was no significant difference between EI and EF plants under low Cd^2+^ concentrations, indicating that *Epichloë* infection was only beneficial to the growth and development of *A. inebrians* and *E. dahuricus* exposed to high Cd^2+^ concentrations.

Studies of nutrient acquisition in EI grasses have focused on the influence of nitrogen (N), since this element is a constituent of alkaloids in infected plants and is also one of the most important limiting resources for plant growth in general (Li et al., 2012a). It has been documented that increased N availability may change the relative availability of other nutrients, such as phosphorus (P) (Van Der Wouder et al., 1994). Li et al. (2012a) found that *A. sibiricum*–*Epichloë* associations are conditional on both N and P availability, but are more conditional on N than P. Changes in N allocation increase the photosynthetic ability of EI plants and also significantly increase their biomass. In addition, the benefits of *Epichloë* infection decline when nutrient availability decreases (Ren et al., 2011). *Epichloë* infection tends to reduce overall nitrogen concentration in *A. sibiricum* leaves, but causes the host to allocate significantly higher fractions of N to the photosynthetic machinery (Ren et al., 2011). Thus, EI plants have higher photosynthetic N use efficiency and shoot biomass than that of EF plants when fertilizer is limited (Ren et al., 2014). Song et al. (2015d) confirmed that EI *H. brevisubulatum* has lower ratios of C:N, C:P, Na^+^:K^+^ and a higher ratio of N:P than EF plants under salt stress. According to Jia et al. (2014), the effects of EI on *A. sibiricum* suggest that the *A*. *sibiricum* host genotype has a stronger influence on the response to stress than the influence of *Epichloë*. They found that *Epichloë* infection did not positively affect general growth, physiology, or nutrient content of *A. sibiricum*, before or after clipping.

### Pest resistance

The grass-*Epichloë* symbiosis provides the grass host protection from herbivorous insects by producing alkaloids in the form of secondary metabolites (García Parisi et al., 2014; Thom et al., 2014). Aphid populations exhibit slow growth when feeding on grass infected with *Epichloë* species (Hartley and Gange, 2009; Saikkonen et al., 2010). However, Börschig et al. (2014) concluded that the effect of *Epichloë* endophytes on herbivores is generally weak and depends on the regional environmental context. They posit that more field research is necessary to detect the relative importance of *Epichloë* endophytes and environmental context on biotic interactions in grasslands (Börschig et al., 2014).

To date, insect resistance has only been reported for *L. chinensis*–*E. bromicola, A. sibiricum*–*E. sibirica* and *A. inebrians*–*E. gansuensis* associations in China. Jia et al. (2013) concluded that *L. chinensis*–*E. bromicola* and *A. sibiricum*–*E. sibirica* symbioses could diminish the negative effects of infection by *Meloidogyne incognita*. The researchers used a 72-h exposure to undiluted culture filtrates of the two endophytes and found *L. chinensis* infected with *E. bromicola* had an especially strong antagonistic effect on *Meloidogyne* infection. Similarly, Zhang et al. (2012b) found that *A. inebrians* infected with *E. gansuensis* reduced the survival of the aphids *Rhopalosiphum padi, Tetranychus cinnabarinus, Oedaleus decorus*, and *Messor aciculatus* under laboratory and field conditions. Additionally, they demonstrate that EI had an anti-herbivore effect on a wide range of arthropod groups (Zhang et al., 2012b).

### Pathogen resistance

Reports that EI grasses are resistant to diseases and pathogens are limited compared to evidence that EI increases pest resistance. *Epichloë* endophytes negatively impact the *in vitro* growth of plant fungal pathogens (White and Cole, 1985; Siegel and Latch, 1991). However, Sabzalian et al. (2012) found that EI tall fescue was not more resistant to powdery mildew (*Blumeria graminis*) than EF tall fescue. Yue et al. (2000) demonstrated that extracts from a wide range of *Epichloë* endophytes exhibited various degrees of antifungal activity and the greatest antifungal activity was detected from extracts of *E. festucae* and *E. tembladerae*.

Li et al. (2007) confirmed that the fungi *Bipolaris sorokiniana, Curvularia lunata, Fusarium acuminatum*, and *Alternaria alternate* cause lesions on detached *A. inebrians* leaves, regardless of their status as EI or EF. When leaves were EF, the number and size of lesions caused by all pathogens were reduced compared to those on EI leaves. In addition, Xia et al. (2015) demonstrated that, in greenhouse conditions, EI reduced the ability of *Blumeria graminis* to colonize *A. inebrians* and enhanced the photosynthetic performance of host plants under pathogen stress or ameliorated host plant damage, to some degree (Xia et al., 2016). Zhou et al. (2015b) found that EI *F. sinensis* produced secondary metabolites that inhibited fungal pathogens, including *Alternaria alternata, Bipolaris sorokiniana, Curvularia lunata*, and *Fusarium acuminatum*. They found significant reductions in disease incidence and lesion size on EI detached leaves compared to EF leaves (Zhou et al., 2015b). Song et al. (2015f) found that *E. bromicola* from *Elymus tangutorum* exhibits antifungal activities against *Alternaria alternata, Fusarium avenaceum, Bipolaris sorokiniana*, and *Curvularia lunata*.

## Molecular identification of chinese *Epichloë* species

In the past, taxonomic identification of *Epichloë* endophytes relied on morphological features, e.g., colony morphology, colony growth rate, and spore type and size. Currently, allozyme profiles and molecular methods have been applied to *Epichloë* research and greatly aid in identification. Recent research combines morphological features and molecular data to identify *Epichloë* endophytes.

*Epichloë* endophytes are typically analyzed using β-tubulin (*tubB*) (Tsai et al., 1994), translation elongation factor 1-α (*tefA*) (Moon et al., 2002), actin (*actG*) (Moon et al., 2007; Zhang et al., 2009), simple sequence repeats (SSR) (Moon et al., 1999; Schirrmann et al., 2015), amplified fragment length polymorphisms (AFLP) (Karimi et al., 2012), internal transcribed spacers of the nuclear ribosomal RNA (ITS) (Moon et al., 2000), calmodulin M (*calM*) (McCargo et al., 2014), and so on. The most common markers for taxon identification and determining phylogenetic relationships are *tubB, tefA*, and *actG* (Clay and Schardl, 2002). These studies have shown that asexual *Epichloë* endophytes evolved from sexual *Epichloë* species and subsequently lost the ability to sexually reproduce (Moon et al., 2000).

Although new *Epichloë* endophytes have been identified based on traditional morphology, this method has limitations when determining whether the *Epichloë* endophytes experienced hybridization events. Fortunately, DNA sequencing can help resolve this problem. To date, all putative *Epichloë* hybrids contain more than one copy of *tubB* and can be detected by allozyme analysis (Moon et al., 2004; Oberhofer and Leuchtmann, 2012; Leuchtmann et al., 2014; Iannone et al., 2015). For example, *E. chisosa* and *E. coenophiala* each have three copies (Leuchtmann et al., 2014), indicating they experienced multiple ancient hybridization events or subsequent gene duplication. Oberhofer and Leuchtmann (2012) found four new *Epichloë* species in *Hordelymus europaeus* using five enzymes; two were interspecific hybrids and the others were of nonhybrid origin.

Molecular markers can be used to identify new species and to estimate evolutionary relationships with phylogenetic trees. Molecular studies on Chinese *Epichloë* species have mainly been applied to identify new species. Various *Epichloë* species, e.g., *E. stromatolonga* (Li et al., 2006b; Ji et al., 2009), *E. sinica* (Kang et al., 2009), *E. sinofestucae* (Chen et al., 2009), *E. liyangensis* (Kang et al., 2011a), and *E*. sp. (Han et al., 2012), have been described and exhibit natural symbioses with *R. kamoji, Calamagrostis epigeios, Roegneria* spp., *F. parvigluma, P. pratensis* ssp. *pratensis*, and *F. myuros*. These *Epichloë* species are native to China and were described based on host specificity, morphology, and molecular phylogenetic evidence. Zhang et al. (2009) identified a new *Epichloë* endophyte, *E. sibirica* (*A. sibiricum*), and three morphotypes based on morphological and phylogenetic analyses. They found that its ancestor was probably derived from *E. sibirica* (Zhang et al., 2009). Zhu et al. (2013) analyzed *L. chinensis* and found that its *Epichloë* associate is *E. bromicola*, which was classified into three morphotypes based on morphological features and phylogenetic analyses of *tubB, tefA*, and *actG* sequences. Additionally, a molecular phylogenetic study showed that *E. gansuensis* var. *inebrians* from Chinese *A. inebrians* is a unique and novel non-hybrid species (Moon et al., 2007).

Although some studies have examined the evolutionary relationships among *Epichloë* species, few have examined the phylogeny or co-evolution of Chinese *Epichloë* species and hosts. In the southern hemisphere, most asexual *Epichloë* species are the result of hybridization events between two sexual species (e.g., *E. festucae* and *E. typhina*) from the northern hemisphere (Gentile et al., 2005). These studies have looked at the extent of *Epichloë* gene flow between the Northern and Southern Hemispheres based on molecular data (Moon et al., 2002). Iannone et al. (2009) studied South American *Epichloë* endophytes from *Bromus auleticus* and found that *E. tembladerae* was a hybrid of the Northern *E. festucae* and *E. typhina*, but the ancestral *E. typhina* genotype was distinguished based on *tubB* and *tefA*. Schirrmann et al. (2015) used 15 microsatellites to assess the population structure of sympatric species in the *E. typhina* complex and found that host specificity and maladaptation of *Epichloë* hybrids to host grasses may act as reproductive isolation barriers in asexual *Epichloë* and therefore promote their speciation.

Notably, Kang et al. (2011b) analyzed the asymptomatic symbiosis between *Roegneria* and *E. sinica* and found no relationship between phylogeny and morphology in the *E. sinica* isolates. They concluded that *E. sinica* is a species complex that resulted from multiple, independent hybridization events (Kang et al., 2011b). In a comparison of genetic diversity in *Epichloë* species and their host plants, Zhang et al. (2010c) found approximately 4–7-fold greater diversity among *Epichloë* endophytes than among host plants based on SSR markers. This indicates more gene flow of *Epichloë* endophytes than hosts. The authors also state that *Epichloë* infection might confer selective advantages to *A. sibiricum* under certain conditions, which could help to maintain high-EI frequencies, even when their population structure would not suggest selection for EI (Zhang et al., 2010c).

Song et al. found that *Epichloë* species likely originated in Eurasia, and *Epichloë* gene flow between the Western and Eastern hemispheres is common based on phylogenetic and network analyses (Song and Nan, 2015; Song et al., 2015a). They suspect that migratory birds or humans might have aided the dispersal of *Epichloë* endophytes from Eurasia to other continents (Song and Nan, 2015). Furthermore, Song et al. (2015c) analyzed *Hordeum*-endophytes and *Elymus*-endophytes and found that Chinese *Hordeum* species likely contain two *Epichloë* endophyte species. One is also found in North American *Elymus* species and the other endophyte is found in Chinese *Elymus* species, indicating that *Epichloë* endophytes isolated from Chinese *Hordeum* are not host-specific. They proposed that *Epichloë* endophytes spread among different grass hosts by plant hybridization, and this could likely transform the hybrid offspring from EF status to EI status (Song et al., 2015c). This needs to be tested in future studies, but it would add further evidence to the hypothesis that asexual *Epichloë* endophytes are horizontally transmitted (Tadych et al., 2012; Wiewióra et al., 2015). Moreover, molecular phylogenetic studies based on *tubB* and *tefA* intron sequences have confirmed that *E. gansuensis* infected *A. sibiricum* and *A. inebrians* in China, indicating the potential of conidia cultures to mediate horizontal transmission (Li et al., 2015).

## Alkaloids

From an agronomic point of view, a negative aspect of the grass-*Epichloë* symbiosis is that some *Epichloë* produce ergot and indole-diterpene fungal alkaloids that are highly toxic for livestock (Clay and Schardl, 2002). Variability in the profile and level of alkaloids has allowed researchers to inoculate grass cultivars with selected *Epichloë* endophytes that are not toxic to livestock and still confer benefits to host plants. This has become a key strategy for breeding drought-, salt-, and pest-resistant forage grasses (Gundel et al., 2013b; Johnson et al., 2013). *A. inebrians* is widely distributed in northern China and is commonly known as drunken horse grass because of its long-recognized toxic and narcotic effects on livestock, especially horses. Additionally, owing to the toxicity to livestock, recent research has shown that *A. inebrians* can protect biodiversity (Yao et al., 2015). These toxins are apparently caused by *E. gansuensis* (Li et al., 2004; Zhang et al., 2014). *Epichloë*-infected drunken horse grass contains high levels of the ergot alkaloids, ergine, and ergonovine (Miles et al., 1996; Li et al., 2006a). High alkaloid levels have also been confirmed in EI *A. inebrians* under salt or drought stress, with higher levels of ergonovine than ergine (Zhang et al., 2011b). Cytotoxic effects to animal muscle tissue have been described after the consumption of ergonovine and ergine (Zhang et al., 2014). The EI *E. dahuricus* only produces the alkaloid peramine. Production is seasonal; the concentration of peramine are highest in October and below detectable levels in June (Zhang and Nan, 2007a). Recently, Zhou et al. (2015a) evaluated the effects of temperature on ergot alkaloid production in three *F. sinensis* ecotypes and found that concentrations of ergine and ergonovine differed considerably in the three endophyte-infected ecotypes. They also found the ecotypes varied in their production of secondary metabolites, the bioprotective alkaloids ergine and ergonovine, in response to short-term cold stress. However, compared to recent research abroad (Schardl et al., 2012), little is known about alkaloid production in Chinese native grasses using molecular methods. We hope to increase research in this area in the future.

## Conclusions and perspectives

In this review, we briefly summarized progress in *Epichloë* endophyte research in China in the past 25 years. We found that more than 77 species of native grasses in China were infected with *Epichloë* species. To date, nine *Epichloë* species have been identified from Chinese native grasses. Additionally, seven have been confirmed as new *Epichloë* endophytes. *Epichloë* species originated in Eurasia based on the high species diversity in the area (Song and Nan, 2015). Unfortunately, many isolates from Chinese native grasses have not been identified to the species level. Therefore, to apply this precious resource, Chinese research should focus on taxonomical evaluations of *Epichloë* species from Chinese native grasses. In addition, Chinese studies have extensively examined abiotic and biotic resistance using *Epichloë* endophytes. However, little is known about *Epichloë* evolution, functional genomics, and comparative genomics. Nevertheless, we believe that Chinese researchers will intensify their efforts in these areas in the future.

## Author contributions

HS wrote the article. ZN served as the principal investigator, facilitated the project, and assisted in manuscript preparation. QS and CX wrote and revised the paper. XL, XY, WX, YK, PT, and QZ explored literature and modified the article.

### Conflict of interest statement

The authors declare that the research was conducted in the absence of any commercial or financial relationships that could be construed as a potential conflict of interest.
